# Country-level income inequality and risky health behaviors of “golden youth” in the post-Communist countries of Europe: A cluster analysis

**DOI:** 10.1016/j.pmedr.2024.102880

**Published:** 2024-09-05

**Authors:** Armen Albert Torchyan, Inge Houkes, Hans Bosma

**Affiliations:** a Department of Social Medicine, Care and Public Health Research Institute (CAPHRI), Faculty of Health, Medicine and Life Sciences, Maastricht University, P.O. Box 616, 6200, MD, Maastricht, the Netherlands; b Department of Family and Community Medicine, College of Medicine and King Khalid University Hospital, King Saud University, P.O. Box, 7805, Riyadh 11472, Saudi Arabia

**Keywords:** Alcohol, Bullying, Golden youth, HBSC, Income inequality, Smoking

## Abstract

**Objective:**

We aim to study the “golden youth” hypothesis, which suggests that risky behaviors might be prevalent among affluent adolescents in post-Communist countries of Europe (PCCE) with high income inequality.

**Methods:**

We included 71,119 adolescents aged 11–15 from 14 PCCE participating in the Health Behavior in School-aged Children survey 2017/18. A K-means cluster analysis was conducted to group adolescents based on risky behavior. Generalized linear mixed models were fitted.

**Results:**

The proportion of high-SEP adolescents in Cluster 2, characterized by frequent alcohol consumption but moderate frequency of drunkenness, was greater than that of low-SEP adolescents (14.3 % vs. 10.7 %). The prevalence of risky behaviors was similar for high and low-SEP adolescents in Cluster 3 (high smoking, frequent alcohol use, drunkenness, and moderate bullying) (8.0 % vs. 8.2 %) and Cluster 4 (high bullying perpetration) (6.7 % vs. 7.2 %). Countries with higher Gini index were at greater risk of reporting risky behaviors. High-SEP adolescents were more likely to engage in risky behaviors in countries with high income inequality. The odds ratios comparing high- vs. low-SEP adolescents ranged from 0.89 in the least unequal to 1.67 in the most unequal countries for multiple risky behaviors (Cluster 3: *P*-interaction = 0.042) and from 0.61 to 1.19 for bullying perpetration (Cluster 4: *P*-interaction = 0.030).

**Conclusions:**

High-SEP adolescents in PCCE might be at increased risk for unhealthy and vicious behaviors, especially in countries with high income inequality. Redistributive policies decreasing the gap between rich and poor are needed to ensure the health and well-being of adolescents in PCCE.

## Highlights

• High-SEP adolescents might be at an increased risk for unhealthy behavior in post-Communist countries of Europe.
• Wide income inequality might contribute to risky behaviors among high-SEP adolescents.
• Policies promoting a fairer distribution of income may be necessary in several post-Communist countries of Europe.

## 1 Introduction

It has been widely recognized that country-level income inequality, as a macro-level, upstream social determinant, considerably impacts population health and behavior (Truesdale and Jencks, 2016). Research has shown that higher levels of income inequality within a country are associated with increased disparities in health, with those from lower-income backgrounds bearing the disproportionate burden of health issues (Truesdale and Jencks, 2016). Notably, similar results have been reported among adolescents, with studies reporting larger socioeconomic differences and worse overall mental well-being in countries with higher income inequality (Dierckens et al., 2020). Factors such as access to healthcare, quality of living conditions, psychosocial stress, and exposure to environmental hazards can all contribute to this relationship (Truesdale and Jencks, 2016). Addressing country-level income inequality is essential for promoting more equitable health outcomes for all members of society.

However, recent studies in post-Communist countries of Europe (PCCE) have found that adolescents from high socioeconomic backgrounds are at an increased risk of unhealthy behaviors, such as frequent alcohol use, drunkenness (Torchyan et al., 2023), and bullying perpetration (Torchyan et al., 2024), especially in countries with large income inequality. Evidence suggests that income inequality can increase status anxiety and related feelings of stress and insecurity (Wilkinson and Pickett, 2011). Similarly, the “golden youth” hypothesis proposed by Torchyan and colleagues (Torchyan et al., 2023, Torchyan et al., 2024) contends that in PCCE with high income inequality some high-SEP adolescents might engage in such unhealthy and vicious behaviors due to the fear of losing their high status, power, and prestige and may try to demonstrate their high standing through behaviors that might be more accepted or that signal high SEP in PCCE, such as alcohol use (Malisauskaite and Klein, 2018) or bullying perpetration (Power et al., 2013).

Unfortunately, there is a lack of research on the “golden youth” in PCCE, extending to other risky health behaviors, such as smoking, or examining multiple risky behaviors. In this study, using data from the Health Behavior in School-aged Children (HBSC) survey, a WHO collaborative study, we aim to examine the risky health behaviors of high-SEP adolescents in PCCE to get a better insight into the potential problem of the “golden youth” across PCCE and the role of income inequality in it. The main objectives of this paper are to 1) identify adolescent clusters with distinct risky health behavior patterns related to tobacco smoking, alcohol use, drunkenness, and bullying perpetration, 2) detect any risky clusters where the proportion of high-SEP adolescents is relatively high, 3) determine whether income inequality increases the likelihood of high-SEP adolescents to be in risky clusters.

## 2 Methods

### 2.1 Study design

In this study, we included 71,119 adolescents aged 11–15 years from 14 PCCE with GDP per capita of more than USD 5,000 (Bulgaria, Croatia, Czechia, Estonia, Hungary, Latvia, Lithuania, Poland, Romania, Russia, Serbia, Slovakia, Slovenia, and North Macedonia) participating in the HBSC study between 2017 and 2018. Country income inequality has been suggested to be the primary determinant of health disparities, particularly when the GDP per capita exceeds USD 5,000 (Wilkinson and Pickett, 2011). The study used a standardized approach in all countries (Inchley J et al., 2018). Nationally representative samples of adolescents were recruited through cluster sampling design, where primary sampling units were classes or schools. Adolescents were given an anonymous self-administered questionnaire to fill out in their classrooms. All procedures were performed in compliance with relevant laws and institutional guidelines and have been approved by the appropriate institutional committees in each participating country. The parents or guardians of all adolescents were fully informed about the research, and all participants provided informed consent. This study was based on a publicly available anonymized database (The HBSC Data Management Centre, 2017). Additional information regarding the methodology has been published elsewhere (Inchley J et al., 2018).

### 2.2 Measures

#### 2.2.1 Risky health behaviors

The smoking status was determined by asking the following question: “On how many days (if any) have you smoked cigarettes in your lifetime.” This method of evaluating adolescent tobacco use has demonstrated 90 % sensitivity and 93 % specificity when compared to the salivary cotinine test for tobacco use (Post et al., 2005). A similar question was asked about alcohol use: “On how many days (if any) have you drunk alcohol in your lifetime?” Response options for both questions were: “never”, “1–2 days”, “3–5 days”, “6–9 days”, “10–19 days”, “20–29 days”, and “30 days (or more).” Drunkenness was assessed using the following question: “Have you ever had so much alcohol that you were really drunk in your lifetime?” with answer categories “No, never”, “Yes, once”, “Yes, 2–3 times”, “Yes, 4–10 times”, “Yes, more than 10 times” (Inchley J et al., 2018). It was found that single questions assessing the frequency of alcohol use among adolescents were highly correlated (Spearman’s Rho = 0.719–––0.90) with the Timeline Follow Back Calendar, which is considered a criterion standard measure of alcohol consumption (Levy et al., 2021). Bullying perpetration was measured by asking students, “How often have you taken part in bullying another person(s) at school in the past couple of months?” using Olweus definition of bullying (Olweus, 1996). Response options were “I have not bullied another person(s) at school in the past couple of months”, “It has happened once or twice”, “2 or 3 times a month”, “About once a week”, Several times a week” (Inchley J et al., 2018). The self-report bullying items used in the HBSC study have demonstrated strong structural validity in measuring bullying among adolescents (Roberson and Renshaw, 2018).

#### 2.2.2 Socioeconomic position

The HBSC Family Affluence Scale (FAS III) was administered to adolescents to assess their family SEP. The participants were presented with a set of questions to answer: 1) “Does your family own a car, van or truck?” (no, one, two or more); 2) “Do you have your own bedroom for yourself?” (no, yes); 3) How many computers does your family own (including laptops and tablets, not including game consoles and smartphones) (none, one, two, more than two); 4) “How many bathrooms (room with a bath/shower or both) are in your home?” (none, one, two, more than two); 5) “Does your family have a dishwasher at home?” (no, yes); 6) “How many times did you and your family travel out of the country for a holiday/vacation last year?” (not at all, once, twice, more than twice) (Inchley J et al., 2018). The responses were added together, with higher scores indicating a higher SEP for the family. Adolescents were categorized into low, middle, and high SEP groups based on country-specific (ridit transformed) FAS III scores (20 %, 60 %, and 20 %, respectively).

#### 2.2.3 Income inequality

The 2017 World Bank statistics were used to obtain estimates for country income inequality measured by the Gini index (World Bank, 2017b). A higher Gini index indicated wider income inequality and was centered at its mean in the models.

#### 2.2.4 Co-variates

In the models, adolescents’ age, sex, bullying victimization, and GDP per capita (World Bank, 2017a) were considered co-variates because they might be associated with tobacco smoking, alcohol use, drunkenness, bullying perpetration, and income inequality. Bullying victimization was assessed by asking the question, “How often have you been bullied at school in the past couple of months?” The response options were the same as those provided for bullying perpetration (see above) (Inchley J et al., 2018).

### 2.3 Statistical analysis

Descriptive statistics were used to characterize the sample. A K-means cluster analysis was conducted to group adolescents based on their risky health behaviors (tobacco smoking, alcohol use, drunkenness, and bullying perpetration). The standardized scores of all variables related to risky health behaviors were employed as continuous variables in the analysis. The Silhouette coefficient was utilized to determine the optimal number of clusters. The coefficient was calculated on a randomly selected 20 % of the total observations to reduce the computational burden associated with the large sample size. A design-adjusted Chi-square test was used to examine the relationship between cluster membership and adolescent age and sex. Generalized linear mixed models were fitted with adolescents (level 1) nested into countries (level 2). A mixed-effects multinomial logistic regression analysis was performed to test the relationship between family socioeconomic position, country income inequality, and cluster membership. In a cross-level interaction analysis, we examined whether income inequality increases the likelihood of being in clusters with risky health behaviors among high-SEP adolescents. This involved introducing a random slope and creating a product term between the Gini index and family SEP. All statistical analyses were performed using IBM SPSS Statistics for Windows, Version 27.0 (IBM Corp., Armonk, NY, USA).

## 3 Results

The study included 71,119 adolescents (49.7 % boys) with a mean age of 13.6 years (standard deviation [SD] = 1.7) from 14 PCCE. The Gini index ranged from 23.2 to 40.4 with the mean of 31.2 (SD=5.4) The country socioeconomic characteristics can be found in Table 1.

**Table 1:** The number of participants and economic characteristics of 14 post-Communist countries of Europe participating in the 2017–18 Health Behavior in School-aged Children survey.

**Countries**	n = 71,119	GDP per capita (USD)	Gini index
Bulgaria	4548	8,366	40.4
Croatia	5169	13,629	30.4
Czechia	11,564	20,636	24.9
Estonia	4725	20,438	30.4
Hungary	3789	14,624	30.6
Latvia	4412	15,695	35.6
Lithuania	3797	16,885	37.3
North Macedonia	4658	5,450	34.2
Poland	5224	13,865	29.7
Romania	4567	10,807	36.0
Russia	4281	10,720	37.2
Serbia	3933	6,293	36.2
Slovakia	4785	17,538	23.2
Slovenia	5667	23,514	24.2

GDP, gross domestic product; note: a higher Gini index denotes greater income inequality.

**Fig. 1:**
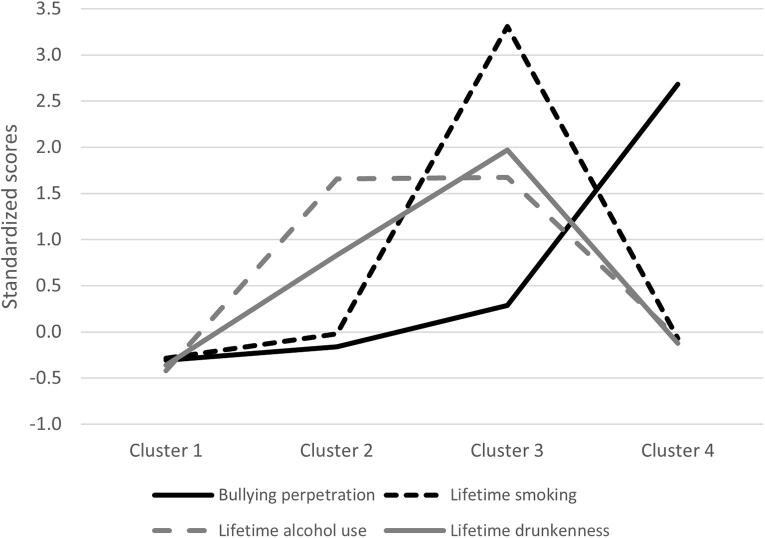
Standardized scores of risky health behaviors among 11 to 15-year-old adolescents from 14 post-Communist countries of Europe participating in the 2017–18 Health Behavior in School-aged Children survey. Note: higher scores indicate more risky health behavior.

Four clusters of adolescents were identified based on their risky health behaviors (see Fig. 1). Demographic characteristics of the four clusters are presented in Table 2. The first cluster of adolescents (73.0 %) displayed the lowest level of risky health behaviors (Fig. 1). The second cluster (12.4 %) was characterized by frequent alcohol consumption but moderate frequency of drunkenness. The third cluster (7.8 %) exhibited multiple risky health behaviors, i.e., high levels of tobacco smoking, frequent alcohol consumption and drunkenness, as well as moderate levels of bullying perpetration. The fourth cluster of adolescents (6.8 %) frequently engaged in bullying perpetration; however, they had low levels of other risky health behaviors.

**Table 2:** Percentages of adolescent characteristics by clusters among 14 post-Communist countries of Europe participating in the 2017–18 Health Behavior in School-aged Children survey (n = 64,435).

**Characteristics**	**Cluster 1**	**Cluster 2**	**Cluster 3**	**Cluster 4**	***P*-value^a^**
	n = 47,014	n = 8,010	n = 5,056	n = 4,355	
Age in years					
11	3.3 %	35.3 %	9.4 %	39.7 %	**< 0.001**
13	18.3 %	39.2 %	27.0 %	36.1 %	
15	78.4 %	25.5 %	63.6 %	24.2 %	
Sex					
Boys	48.1 %	61.4 %	53.6 %	47.3 %	**< 0.001**
Girls	51.9 %	38.6 %	46.4 %	52.7 %	

Bold values denote statistical significance (*P*<0.05); a: a design-adjusted Chi-square test was used; Cluster 1: low risky health behaviors; Cluster 2: frequent alcohol consumption but moderate frequency of drunkenness; Cluster 3: high tobacco smoking, frequent alcohol consumption and drunkenness, moderate bullying perpetration; Cluster 4: high bullying perpetration.

**Fig. 2:**
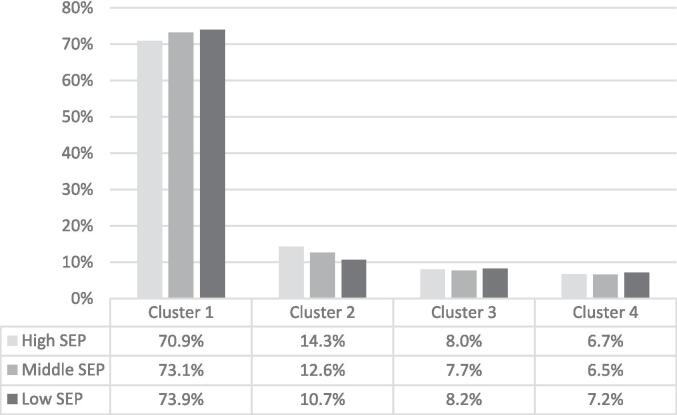
The percentage of adolescents in each cluster by family socioeconomic position (SEP), measured by the Family Affluence Scale (FAS III), among 11 to 15-year-old adolescents from 14 post-Communist countries of Europe participating in the 2017–18 Health Behavior in School-aged Children survey. Cluster 1: low risky health behaviors; Cluster 2: frequent alcohol consumption but moderate frequency of drunkenness; Cluster 3: high tobacco smoking, frequent alcohol consumption and drunkenness, moderate bullying perpetration; Cluster 4: high bullying perpetration.

The percentage of high-SEP adolescents with low risky health behaviors (Cluster 1) was slightly lower compared to middle and low-SEP groups (70.9 % vs. 73.1 % vs. 73.9 %, respectively) (Fig. 2). The proportion of high-SEP adolescents in the second cluster was greater compared to those from lower-SEP families (14.3 % vs. 12.6 %. vs. 10.7 %). The prevalence of risky behaviors was similar between high and low-SEP adolescents in Cluster 3 (8.0 % vs. 7.7 %. vs. 8.2 %) and Cluster 4 (6.7 % vs. 6.5 % vs. 7.2 %).

**Fig. 3:**
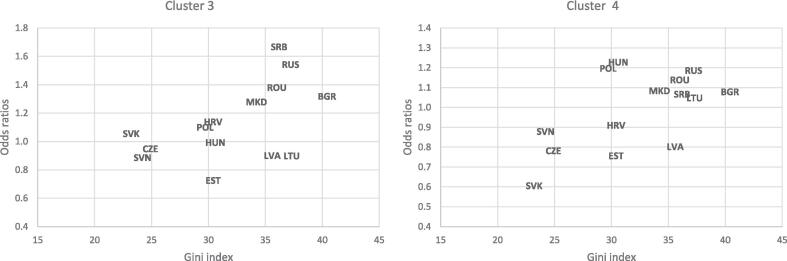
Odds ratios of being in Clusters 3 and 4 (vs. Cluster 1) by family socioeconomic position (high 20% vs. low 20%), adjusted for age and sex, among 11 to 15-year-old adolescents from 14 post-Communist countries of Europe participating in the 2017–18 Health Behavior in School-aged Children survey. Notes: the models were not adjusted for GDP per capita; a higher Gini index denotes greater income inequality. BGR, Bulgaria; CZE, Czechia; EST, Estonia; HRV, Croatia; HUN, Hungary; LTU, Lithuania; LVA, Latvia; MKD, North Macedonia; POL, Poland; ROU, Romania; RUS, Russia; SRB, Serbia; SVK, Slovakia; SVN, Slovenia. Cluster 1: low risky health behaviors; Cluster 3: high tobacco smoking, frequent alcohol consumption and drunkenness, moderate bullying perpetration; Cluster 4: high bullying perpetration.

In a multivariate analysis (Table 3), high-SEP adolescents had 1.5-fold higher odds of frequent alcohol consumption but moderate frequency of drunkenness than their peers from low-SEP families (Cluster 2: *P*<0.001). Multiple risky health behaviors (Cluster 3: *P*=0.300) and bullying perpetration (Cluster 4: *P*=0.385) were not statistically significantly different between high and low-SEP adolescents. Countries with higher Gini index were at a greater risk of reporting multiple risky health behaviors (Cluster 3: *P*=0.107) and bullying perpetration (Cluster 4: *P*<0.001), although the relationship between income inequality and Cluster 3 was not statistically significant. High-SEP adolescents were more likely to engage in risky health behaviors in countries with high income inequality (Table 3 and Fig. 3). The odds ratios comparing high- vs. low-SEP adolescents ranged from 0.89 in the least unequal to 1.67 in the most unequal countries for multiple risky health behaviors (Cluster 3; *P*-interaction = 0.042), and from 0.61 to 1.19 for bullying perpetration (Cluster 4; *P*-interaction = 0.030).

**Table 3:** Odds ratios (95 % confidence intervals) of being in clusters with risky health behaviors by family socioeconomic position and country income inequality, adjusted for age, sex, and GDP per capita, among 11 to 15-year-old adolescents from 14 post-Communist countries of Europe participating in the 2017–18 Health Behavior in School-aged Children survey (n = 63,636).

**Characteristics**	**Cluster 1**	**Cluster 2**	**Cluster 3**	**Cluster 4**
Family SEP^a^				
High	Reference	1.51 (1.39 – 1.64)	1.05 (0.95 – 1.16)	0.96 (0.86 – 1.06)
Middle	Reference	1.23 (1.15 – 1.32)	0.95 (0.88 – 1.03)	0.90 (0.83 – 0.98)
Low	Reference	Reference	Reference	Reference
Gini index^a^	Reference	1.00 (0.92 – 1.09)	1.06 (0.99 – 1.13)	1.11 (1.05 – 1.17)
Cross-level interaction				
Gini index*high SEP	Reference	0.98 (0.97 – 1.00)	1.02 (1.00 – 1.04)	1.02 (1.00 – 1.04)
Gini index*middle SEP	Reference	1.00 (0.98 – 1.01)	1.02 (1.00 – 1.04)	1.03 (1.01 – 1.04)

SEP, socioeconomic position; a: the results presented are from a model that does not include interaction terms. Cluster 1: low risky health behaviors; Cluster 2: frequent alcohol consumption but moderate frequency of drunkenness; Cluster 3: high tobacco smoking, frequent alcohol consumption and drunkenness, moderate bullying perpetration; Cluster 4: high bullying perpetration.

## 4 Discussion

Using nationally representative samples of 71,119 adolescents participating in the HBSC survey, this study provides valuable insights into socioeconomic differences in risky health behaviors of adolescents in PCCE. The findings suggest that adolescents from high socioeconomic backgrounds in PCCE might be at an increased risk for unhealthy behaviors, especially in countries with high income inequality.

Our findings support the presence of “golden youth” in PCCE, and particularly in the most unequal countries. The term “golden youth” is used in PCCE to describe the “privileged” children of affluent families who exhibit high levels of fun-seeking and irresponsible behavior (SCHIMPFÖSSL, E., 2018, Baker et al., 2007). In a study by Schelleman-Offermans and colleagues (Schelleman-Offermans et al., 2022), socializing and enhancement motives mediated the relationship between high SEP and increased drunkenness among adolescents in PCCE. Simetin et al. (Simetin et al., 2011) suggested that Croatian adolescents from high socio-economic backgrounds may use alcohol as a symbol of their high social standing. Therefore, it is possible that, as the “golden youth hypothesis suggests, some adolescents from high socio-economic backgrounds might feel an increased need to demonstrate their high social standing and resort to the abovementioned risky health behaviors when other avenues are not available. As Wilkinson argues (Wilkinson and Pickett, 2011), this status anxiety is more likely to be greater in countries with high income inequality.

Our study has some limitations that need to be acknowledged. Adolescents, particularly those from high-SEP families, may underreport risky health behaviors to provide more socially desirable answers, leading to an underestimation of the overall prevalence of risky health behaviors and the magnitude of associations. The limited number of level 2 units (14) may have impacted the power of detecting differences and the strength thereof, related to country-level variables. Nevertheless, given the relatively small effect size, it is important to assess the practical significance of the findings critically. Finally, the proportion of missing values was low (4.6 % or less) (see Table S1) and did not follow any regular patterns. Therefore, it is unlikely that missing values influenced our results.

In conclusion, this study provides valuable evidence that high-SEP adolescents in PCCE may be vulnerable to risky behaviors, particularly in countries with high income inequality. Our findings highlight the need for redistributive policies decreasing the gap between rich and poor to ensure the health and well-being of adolescents across socioeconomic strata in PCCE. Further studies, including ones with a qualitative design, are needed to better understand the complex relationship between income inequality, SEP, and risky health behaviors of adolescents in PCCE and to explore in more depth whether status anxiety is the underlying cause of increased substance use among the “golden youth.”.

## Funding

This research did not receive any specific grant from funding agencies in the public, commercial, or not-for-profit sectors.

## CRediT authorship contribution statement

**Armen Albert Torchyan:** Writing – original draft, Visualization, Methodology, Formal analysis, Data curation, Conceptualization. **Inge Houkes:** Writing – review & editing, Visualization, Supervision, Methodology, Conceptualization. **Hans Bosma:** Writing – review & editing, Visualization, Supervision, Methodology, Conceptualization.

## Declaration of competing interest

The authors declare that they have no known competing financial interests or personal relationships that could have appeared to influence the work reported in this paper.

## Data Availability

The data that support the findings of this study are available in the HBSC Data Management Centre at https://www.uib.no/en/hbscdata/113290/open-access
