# Supplementary material for: Country-level income inequality and risky health behaviors of “golden youth” in the post-Communist countries of Europe: A cluster analysis
Source: Prev Med Rep. 2024 Sep 5;46:102880. doi: 10.1016/j.pmedr.2024.102880 (PMC11406008; doi:10.1016/j.pmedr.2024.102880)
Supplement: Supplementary Data 1 [file mmc1.docx]

| **Table S1**. The number and percentage of missing values among 11 to 15-year-old adolescents from 14 post-Communist countries of Europe participating in the 2017-18 Health Behavior in School-aged Children survey (n = 71,119) | | |
| --- | --- | --- |
| **Characteristics** | **Missing** | |
|  | n | % |
| Age | 143 | 0.2 |
| Sex | 0 | 0.0 |
| Family SEP | 3267 | 4.6 |
| Bullying perpetration | 1994 | 2.8 |
| Lifetime smoking | 3179 | 4.5 |
| Lifetime alcohol use | 2812 | 4.0 |
| Lifetime binge drinking | 1947 | 2.7 |
| Gini index | 0 | 0.0 |
| GDP per capita | 0 | 0.0 |

SEP, socioeconomic position
